# Pneumococcal BgaA Promotes Host Organ Bleeding and Coagulation in a Mouse Sepsis Model

**DOI:** 10.3389/fcimb.2022.844000

**Published:** 2022-07-01

**Authors:** Moe Takemura, Masaya Yamaguchi, Momoko Kobayashi, Tomoko Sumitomo, Yujiro Hirose, Daisuke Okuzaki, Masayuki Ono, Daisuke Motooka, Kana Goto, Masanobu Nakata, Narikazu Uzawa, Shigetada Kawabata

**Affiliations:** ^1^ Department of Oral and Molecular Microbiology, Osaka University Graduate School of Dentistry, Suita, Osaka, Japan; ^2^ Department of Oral and Maxillofacial Surgery II, Osaka University Graduate School of Dentistry, Suita, Osaka, Japan; ^3^ Genome Information Research Center, Research Institute for Microbial Diseases, Osaka University, Suita, Osaka, Japan; ^4^ Department of Pediatric Dentistry, Okayama University Graduate School of Medicine, Dentistry and Pharmaceutical Sciences, Okayama, Japan; ^5^ Department of Oral Microbiology, Kagoshima University Graduate School of Medical and Dental Sciences, Kagoshima, Japan

**Keywords:** *Streptococcus pneumoniae*, BgaA, coagulation, virulence factor, neutrophil

## Abstract

*Streptococcus pneumoniae* is a major cause of invasive diseases such as pneumonia, meningitis, and sepsis, with high associated mortality. Our previous molecular evolutionary analysis revealed that the *S. pneumoniae* gene *bgaA*, encoding the enzyme β-galactosidase (BgaA), had a high proportion of codons under negative selection among the examined pneumococcal genes and that deletion of *bgaA* significantly reduced host mortality in a mouse intravenous infection assay. BgaA is a multifunctional protein that plays a role in cleaving terminal galactose in *N*-linked glycans, resistance to human neutrophil-mediated opsonophagocytic killing, and bacterial adherence to human epithelial cells. In this study, we performed *in vitro* and *in vivo* assays to evaluate the precise role of *bgaA* as a virulence factor in sepsis. Our *in vitro* assays showed that the deletion of *bgaA* significantly reduced the bacterial association with human lung epithelial and vascular endothelial cells. The deletion of *bgaA* also reduced pneumococcal survival in human blood by promoting neutrophil-mediated killing, but did not affect pneumococcal survival in mouse blood. In a mouse sepsis model, mice infected with an *S. pneumoniae bgaA*-deleted mutant strain exhibited upregulated host innate immunity pathways, suppressed tissue damage, and blood coagulation compared with mice infected with the wild-type strain. These results suggest that BgaA functions as a multifunctional virulence factor whereby it induces host tissue damage and blood coagulation. Taken together, our results suggest that BgaA could be an attractive target for drug design and vaccine development to control pneumococcal infection.

## Introduction

*Streptococcus pneumoniae* is an α-hemolytic gram-positive bacterium. Approximately 5%–10% of healthy adults and 20%–40% of healthy children carry *S. pneumoniae* asymptomatically in their oral and nasopharyngeal mucosa (Bogaert et al., 2004; Otsuka et al., 2013). Nonetheless, pneumococcal pneumonia is responsible for approximately 200 million cases and ~1.2 million deaths annually worldwide (GBD 2016 LowerRespiratory Infections Collaborators). In addition, *S. pneumoniae* can invade the host bloodstream, grow in blood and/or organs, and cause invasive pneumococcal diseases (IPD) such as meningitis and sepsis (CDC, 2019).

Currently, pneumococcal vaccines such as the 13-valent pneumococcal conjugate vaccine (PCV-13) and the 23-valent pneumococcal polysaccharide vaccine (PPV23) are licensed and used in many countries. Although pneumococcal vaccines have significantly reduced the incidence of pneumococcal infections globally (CDC, 2019), they also create a selective pressure that increases the emergence of non-vaccine serotypes of *S. pneumoniae*, causing the incidence of IPD to remain substantial (Flasche et al., 2011; Golubchik et al., 2012; Kim et al., 2016; Asner et al., 2019). In addition, *S. pneumoniae* is an antibiotic-resistant medium-priority pathogen, and antibiotic-resistant pneumococcal clones are emerging and expanding (WHO, 2017; CDC, 2019). Recently, we reported that pneumococcal strains isolated in Yangon, Myanmar, carried various antimicrobial resistance genes (Yamaguchi et al., 2021) that hinder the treatment of IPD. Thus, understanding the pneumococcal infectious process and the exacerbation mechanism is necessary to control IPD.

Previously, we performed molecular evolutionary analysis and laboratory-based analyses of streptococcal proteins (Yamaguchi et al., 2016; Yamaguchi, 2018; Yamaguchi et al., 2019a; Yamaguchi et al., 2019b). Negative selection plays an important role in maintaining the long-term stability of biological structures by removing deleterious mutations (Loewe, 2008). Mutations in nonessential but important genes promote the selection of bacterial lineages in the species. In other words, nonessential genes are under considerable negative selection, which would be important for the survival and/or evolutionary success of the species in the host and/or the environment. Our previous study focused on the evolutionary selective pressure on cell wall-anchoring proteins and demonstrated that *bgaA* was under remarkable negative selection pressure (Yamaguchi et al., 2020). The *bgaA* gene encodes the enzyme exo-β-galactosidase (BgaA), which hydrolyzes β1-4 linkages between galactose and glucose or *N*-acetylglucosamine residues (Hobbs et al., 2018). Further bioinformatic and *in vitro* analyses indicated that the BgaA active site is evolutionarily conserved, and that the deletion of *bgaA* in *S. pneumoniae* significantly reduced mortality in a mouse model of blood infection (Yamaguchi et al., 2020). However, the precise role of BgaA in pneumococcal sepsis remains unknown.

*S. pneumoniae* expresses surface-associated exoglycosidases, including BgaA, the exo-α-neuraminidase NanN, and the exo-β-*N*-acetylglucosaminidase StrH, which can degrade some host *N*-glycans (Robb et al., 2017). This results in the release of glycan molecules, which can be utilized by *S. pneumoniae* as a carbon source (Robb et al., 2017). In addition to its galactosidase activity, BgaA presents additional roles and is regarded as a multifunctional protein. For example, an adhesion assay using human epithelial cell lines showed that BgaA contributes to pneumococcal adherence to the cells in a glycosidase activity-independent manner (Limoli et al., 2011). BgaA also contributes to *in vivo* biofilm formation through the exposure of galactose (Blanchette et al., 2016). In addition, an opsonophagocytic killing assay indicated that BgaA plays an important role in the resistance to complement deposition and subsequent phagocytic killing (Dalia et al., 2010). In this study, we performed several *in vitro* and *in vivo* infection assays to further elucidate the role of BgaA in sepsis.

## Materials and Methods

### Bacterial Strains and Cell Culture

*S. pneumoniae* strains were cultured as previously described elsewhere (Mori et al., 2012; Yamaguchi et al., 2019b; Yamaguchi et al., 2020). Briefly, the *S. pneumoniae* strain TIGR4 wild type (WT) and its isogenic *bgaA* mutant strain [Δ*bgaA*, (Yamaguchi et al., 2020)] were cultured at 37°C in Todd-Hewitt broth (BD Biosciences, Franklin Lakes, NJ, USA) supplemented with 0.2% yeast extract (THY; BD Biosciences). For the following assays, *S. pneumoniae* strains were grown to exponential growth phase (OD_600_ = ~0.40) unless otherwise indicated and then resuspended in phosphate buffered saline (PBS) or the appropriate buffer.

Cell culture was performed as previously described (Sumitomo et al., 2012; Yamaguchi et al., 2016). Briefly, human alveolar A549 cells were maintained in Dulbecco’s modified Eagle’s medium (DMEM; Fujifilm Wako Pure Chemical Corporation, Osaka, Japan) supplemented with 10% fetal bovine serum (FBS), and human brain endothelial cells (hBMECs) were maintained in RPMI 1640 medium (Fujifilm Wako Pure Chemical Corporation) supplemented with 10% FBS, 10% NuSerum (BD Biosciences), and 1% MEM nonessential amino acids (Merck KGaA, Darmstadt, Germany). hBMECs were seeded and grown in collagen-coated plates. All cells were maintained at 37°C in a 5% CO_2_ humidified environment.

### *S. pneumoniae* Association Assays

Pneumococcal association with A549 cells and hBMECs was performed as previously described, with minor modifications (Yamaguchi et al., 2008; Yamaguchi et al., 2017). Briefly, human epithelial or endothelial cells were seeded at a density of 1 × 10^5^ cells per well in 24-well plates 24 h before infection. In each well, 0.3–6.0 × 10^7^ CFU of *S. pneumoniae* was added to infect cells. To determine the bacterial association, the infected cells were incubated for 1 h, washed twice with PBS, and then harvested with a solution containing 0.05% trypsin and 0.025% Triton X-100. The associated *S. pneumoniae* was quantified using serial dilution plating on THY-blood agar.

### Blood and Neutrophil Bactericidal Assays

Bactericidal assays were performed as previously described, with minor modifications (Mori et al., 2012; Yamaguchi et al., 2019a; Yamaguchi et al., 2019b). Briefly, heparinized human blood (190 µL), mouse blood (180 μL), or human neutrophils (2 × 10^5^ cells in 180 μL), and exponential phase bacteria (0.9–2.0 × 10^4^ CFU, 1.5–13.3 × 10^4^ CFU, and 1.8–8.3 × 10^4^ CFU for human blood, mouse blood, and human neutrophils in 10, 20, or 20 μL of PBS, respectively) were mixed in 96-well plates and incubated at 37°C in 5% CO_2_ for 1, 2, or 3 h. Viable cell counts were determined by plating diluted samples on THY-blood agar. The growth index was calculated as the number of CFUs at the specified time point divided by the number of CFUs in the initial inoculum.

Human blood was collected *via* the median cubital vein from healthy donors who agreed to a protocol approved by the Institutional Review Board of Osaka University Graduate School of Dentistry (H26-E43). Human neutrophils were isolated from fresh human blood using Polymorphprep (Alere Technologies AS, Oslo, Norway) according to the manufacturer’s instructions. Mouse blood was collected *via* cardiac puncture from healthy 6–7 weeks-old CD-1 female mice (Slc:ICR; Japan SLC, Hamamatsu, Japan). All mouse experiments were conducted following a protocol approved by the Animal Care and Use Committee of Osaka University Graduate School of Dentistry (28-002-0).

### Mouse Infection Assays

Mouse infection assays were performed as previously described (Hirose et al., 2018; Yamaguchi et al., 2019a; Yamaguchi et al., 2019b; Yamaguchi et al., 2020). In the sepsis model, female CD-1 mice (Slc:ICR, 6–7-weeks-old) were intravenously infected with 0.4–7.1 ×  10^6^ CFU of *S. pneumoniae* TIGR4 WT or Δ*bgaA* strains *via* the tail vein. To assess bacterial burden, animals were euthanized by lethal intraperitoneal injection of sodium pentobarbital 24  and 36 h after intravenous infection, and blood, brain, lung, liver, spleen, and kidney samples were collected. Bacterial counts in blood or tissue homogenates were determined after plating serial dilutions, with those in the organ corrected for differences in tissue weight. To examine the histopathological features, each tissue specimen was fixed with 4% formaldehyde, embedded in paraffin, and cut into sections that were stained with hematoxylin and eosin solution. The stained tissues were observed using a BZ-X710 microscope (Keyence, Osaka, Japan).

### RNA-Seq and Data Analysis

Murine blood was obtained at 12, 24, and 36 h after intravenous infection by cardiac puncture after lethal intraperitoneal injection of sodium pentobarbital. Heparin was added to the blood at a final concentration of 30 U/mL. Total RNA was extracted from leukocytes in the whole blood samples using the PureLink Total RNA Blood Purification Kit and DNase I, Amplification Grade (Thermo Fisher Scientific). RNA integrity was assessed using a 2100 Bioanalyzer (Agilent Technologies, Santa Clara, CA, USA). Full-length cDNA was generated using a SMART-Seq HT Kit (Takara Bio, Shiga, Japan) according to the manufacturer’s instructions. An Illumina library was prepared using a Nextera XT DNA Library Prep Kit (Illumina, San Diego, CA, USA) according to SMARTer kit instructions. Libraries were sequenced using the Illumina HiSeq 2500 system, with 75 bp single-end reads obtained. The obtained RNA-seq data were analyzed using iDEP ver 0.91, 0.92 or 0.951 (Ge et al., 2018; Ge, 2021). Pathway analysis was performed using the GAGE package and Kyoto Encyclopedia of Genes and Genomes (KEGG) or Reactome datasets on iDEP (Yu and He, 2016; Kanehisa et al., 2017). Raw data generated in this study was submitted to the Gene Expression Omnibus (GEO) dataset under the accession number GSE190418.

### Measurement of Mouse Plasma Cytokines and Chemokines

Mouse plasma was collected at 24 and 36 h after pneumococcal intravenous infection. Cytokine and chemokine concentrations in mouse plasma were measured by MILLIPLEX MAP Mouse Cytokine/Chemokine Magnetic Bead Panel (catalogue number: MCYTOMAG-70K, Merck) and MAGPIX Dx instrument with xPONENT 4.2 (Luminex Japan, Tokyo, Japan) according to the manufacturers’ instructions. Cytokine and chemokine concentrations were inferred from the standards by the acquisition software. We excluded several cytokines and chemokines for which the bead counts were insufficient or several values exceeded the measurement limits from the final data, which precluded accurate statistical analysis. Heatmap was generated using R software ver. 4.1.2 with gplots package.

### Blood Coagulation Test and Platelet Aggregation Assay

Mice were euthanized by lethal intraperitoneal injection of sodium pentobarbital 36 h after intravenous infection, and blood aliquots were immediately collected. Blood was drawn from the vena cava of the heart into a syringe containing 3.2% (w/v) sodium citrate. Blood samples were centrifuged for 10 min at 845 × *g* at room temperature, and the plasma was collected. Prothrombin time and fibrinogen levels were measured using a semi-automated coagulation analyzer KC1 Delta (Tcong Ireland Ltd., Wicklow, Ireland) and the reagents of prothrombin time and fibrinogen (Sysmex, Hyogo, Japan) according to manufacturer’s guidelines (Nakahara et al., 2013).

For platelet aggregation assay, platelet-rich plasma (PRP) was prepared by centrifugation of anticoagulated whole blood at room temperature (104 × *g*) for 10 min. The blood remaining after removing the PRP was centrifuged at 845 × *g* for 10 min at room temperature to collect platelet-poor plasma (PPP). To measure platelet aggregation of infected mice, the plasmas were collected at 36 h after intravenous infection. For platelet aggregation assay *in vitro*, the plasmas were collected from uninfected mice. Obtained PRP was incubated for 30 min at room temperature with *S. pneumoniae* TIGR4 strains (1.5-2.5 × 10^6^ CFU) WT, Δ*bgaA*, or Δ*bgaA* with 40 units of commercial recombinant BgaA (rBgaA; β1-4 Galactosidase S, New England Biolabs Japan Inc., Tokyo, Japan). Platelet aggregation was assessed by light transmission at 37°C using a PRP3000S device (TAIYO Instruments, Inc., Osaka, Japan) according to the manufacturer’s instructions. Platelets were tested for normal responses to collagen (10 μg/mL). The change in light transmission was recorded and compared with that of autologous PPP, which was considered 100% of light transmission.

### Statistical Analysis

Statistical analysis of the data obtained from the experiments was performed using the Mann–Whitney *U*-test or Kruskal–Wallis test with Dunn’s multiple comparisons test. Differences were considered statistically significant at *P* < 0.05. The tests were carried out using Graph Pad Prism version 7.0d or 8.4.2 (GraphPad Software, Inc., San Diego, CA, USA).

## Results

### BgaA Contributes to Pneumococcal Association of Human Epithelial Cells and Vascular Endothelial Cells

**Figure 1:**
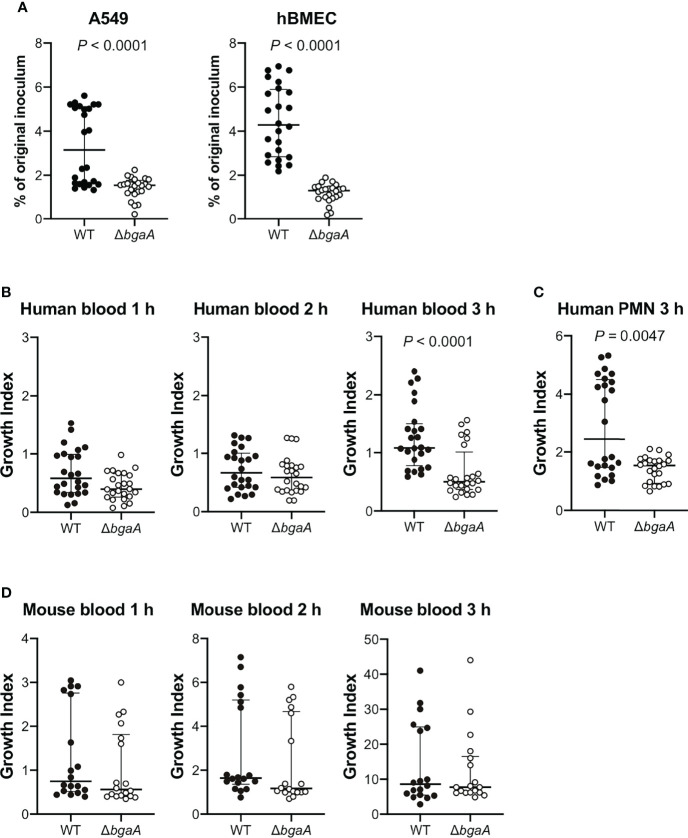
Deficiency of *bgaA* decreases pneumococcal association with human epithelial and endothelial cells and survival after incubation with human neutrophils. **(A)** Rate of association with A549 cells and hBMECs by *S. pneumoniae* TIGR4 wild type and Δ*bgaA* strains. Association rates were calculated by dividing the CFU value obtained at one hour after infection by the value for the original inoculum. **(B)** Growth index of *S. pneumoniae* WT and Δ*bgaA* strains in human blood. Bacterial cells were incubated in human blood for one, two, and three hours at 37°C. **(C)** Growth index of *S. pneumoniae* WT and Δ*bgaA* strains with human neutrophils. Bacterial cells were incubated with human neutrophils for 3 h at 37°C. **(D)** Growth index of *S. pneumoniae* WT and Δ*bgaA* strains in mouse blood. Bacterial cells were incubated in mouse blood for one, two, and three hours at 37°C. **(B–D)** After incubation, samples were serially diluted and plated on TS blood agar. The number of CFUs was determined following incubation. Growth index was calculated by dividing the CFU after incubation by the CFU of the original inoculum. The median and interquartile range (IQR) are represented using horizontal and vertical lines. Differences between groups were analyzed using Mann–Whitney *U*-test. The data were pooled from three or four independent experiments. Individual values are provided in **Supplementary Data 1**.

The interaction between *S. pneumoniae* and the epithelial cells of its host is a prerequisite for pneumococcal disease development. Previous reports have demonstrated that BgaA contributes to the adherence of pneumococcal strains to human epithelial cells. The *bgaA* mutants in R6 and D39 presented reduced adherence to several epithelial cell lines, such as the human pharyngeal carcinoma cell line Detroit-562 and the human lung carcinoma cell line A549 cells (Limoli et al., 2011; Singh et al., 2014). However, the interaction between pneumococcal BgaA and human endothelial cells has not been previously reported. We confirmed the role of BgaA in pneumococcal association with A549 cells and investigated whether BgaA contributes to pneumococcal association with hBMECs. The Δ*bgaA* strain showed significantly lower rates of association with A549 and hBMECs compared with the WT strains (**Figure 1A**). These results suggest that BgaA contributes to pneumococcal association with endothelial cells and epithelial cells *in vitro*.

### BgaA Promotes Pneumococcal Resistance to Human Neutrophil Killing

BgaA inhibits complement-mediated opsonophagocytosis (Dalia et al., 2010). To investigate whether BgaA contributes to pneumococcal survival in blood, we performed bactericidal assays using human blood. After one and two hours of incubation in human blood, there was no significant difference between the survival rates of the WT and Δ*bgaA* strains. However, after three hours of incubation, the survival rate of Δ*bgaA* strains was significantly decreased compared with that of WT strains (**Figure 1B**). In addition, we determined the pneumococcal survival rate after incubation for three hours with human neutrophils. The Δ*bgaA* strain had a significantly lower survival rate than that of the WT after three hours of incubation with human neutrophils, in line with the results of the human blood bactericidal assay (**Figure 1C**). Next, we performed a mouse blood bactericidal assay. In contrast to the effect observed after incubation with human blood, there were no significant differences between the survival of WT and Δ*bgaA* strains after incubation with mouse blood (**Figure 1D**). Previously, we have shown no large difference in the growth curve of WT and Δ*bgaA* strains in the THY medium (Yamaguchi et al., 2020). Altogether, these data indicate that BgaA contributes to pneumococcal human-specific resistance to neutrophil killing under a condition without serum components as well as to the evasion from complement-mediated opsonophagocytosis (Dalia et al., 2010).

### BgaA Deficiency Decreases Tissue Damages in a Mouse Sepsis Model

**Figure 2:**
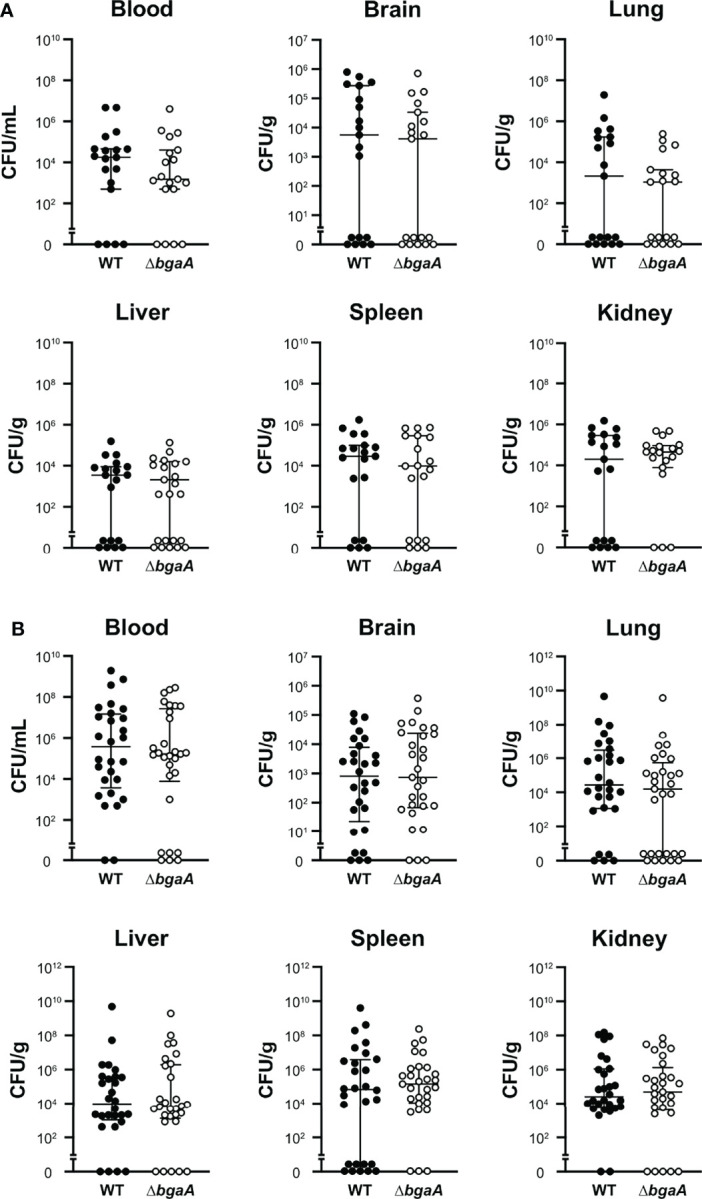
Deficiency of *bgaA* did not affect the bacterial burden in mice. Mice were infected by intravenous injection of *S. pneumoniae* TIGR4 wild type or Δ*bgaA.* The bacterial burden in the blood, brain, lung, liver, spleen, and kidney was assessed after 24 h **(A)** or 36 h **(B)** of infection. The median and IQR values are represented using vertical lines. All mice were perfused with PBS after blood collection, and organ samples were collected. Statistical differences between groups were analyzed using the Mann–Whitney U test. The bacterial burden values obtained from the four independent experiments were pooled. Individual values are provided in **Supplementary Data 2**.

**Figure 3:**
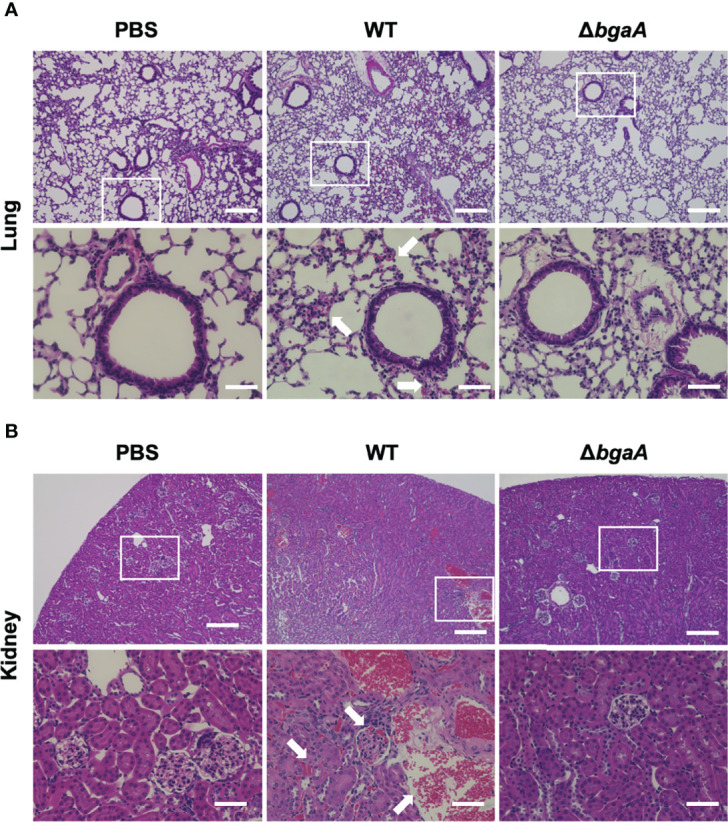
Deficiency of *bgaA* decreases bleeding and microthrombus formation in mouse organs. Haematoxylin and eosin staining of infected mouse lungs **(A)** and kidneys **(B)** collected at 36 h after intravenous infection with PBS or *S. pneumoniae* TIGR4 WT or Δ*bgaA* strains. Arrows indicate bleedings and/or microthrombus formations. Scale bars are 200 µm (upper panels) and 40 µm (lower panels).

Our previous study indicated that the virulence of the Δ*bgaA* strain was significantly lower than that of the WT strain in a mouse sepsis model (Yamaguchi et al., 2020). To elucidate the precise role of BgaA in sepsis, we intravenously infected mice with pneumococcal strains and compared the bacterial burden in the blood, brain, lung, liver, spleen, and kidney 24 and 36 h after infection. In line with the results of previous studies, some of the mice infected with WT *S. pneumoniae* showed signs of imminent death 36 h after infection (Yamaguchi et al., 2020). At 24 h and 36 h after infection, the amount of CFUs in the blood, lung, liver, spleen, and kidney samples of the WT and Δ*bgaA* strains were similar (**Figure 2**). We also examined the histopathological features of the lungs and kidneys, as sepsis often leads to impaired function of these key organs (Fink and Warren, 2014). Histopathological analysis was performed in a blinded fashion by an independent pathologist. Microscopic observation of lungs and kidney samples stained with hematoxylin and eosin revealed that WT*-*infected mice exhibited more bleeding than PBS-treated or Δ*bgaA-*infected mice (**Figure 3**). Moreover, WT*-*infected mice showed bleeding in the renal bodies in the kidney, and microthrombi in the lungs and kidney, in addition to bleeding. Taken together, these results indicate that the deletion of *bgaA* reduces tissue damage in infected mice and decreases pneumococcal pathogenicity in a mouse sepsis model.

**Figure 4:**
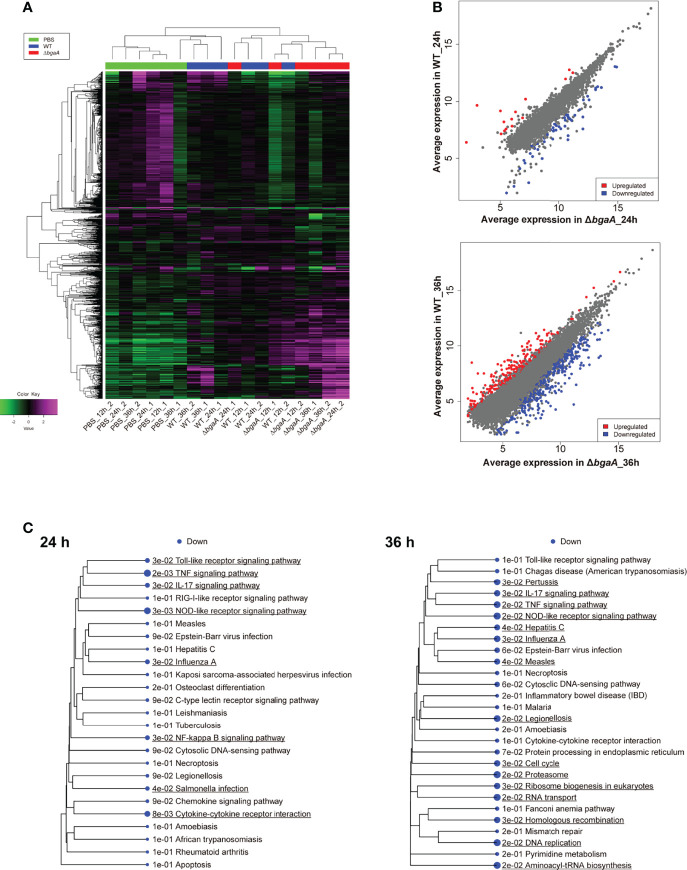
Mouse blood RNA-seq analysis revealed that deficiency of *bgaA* up-regulated several innate immune pathways. RNA-seq analysis was performed using mouse blood at 12, 24 and 36 h after infection. All analysis and visualizations were performed using the iDEP package. **(A)** Heatmap with hierarchical clustering of most variable 1,000 genes. The data is centered by subtracting the average expression level for each gene. **(B)** Scatter plots illustrating host genes consistently altered among WT-infected and Δ*bgaA*-infected mice 24 and 36 h after infection. Differentially expressed genes were calculated using DESeq2. FDR cutoff value was 0.1 and Min fold change was 2. **(C)** Pathway trees. Enrichment pathway analyses were performed by GAGE using KEGG pathways. Circle sizes represent FDR values. FDR cutoff value was 0.2.

**Figure 5:**
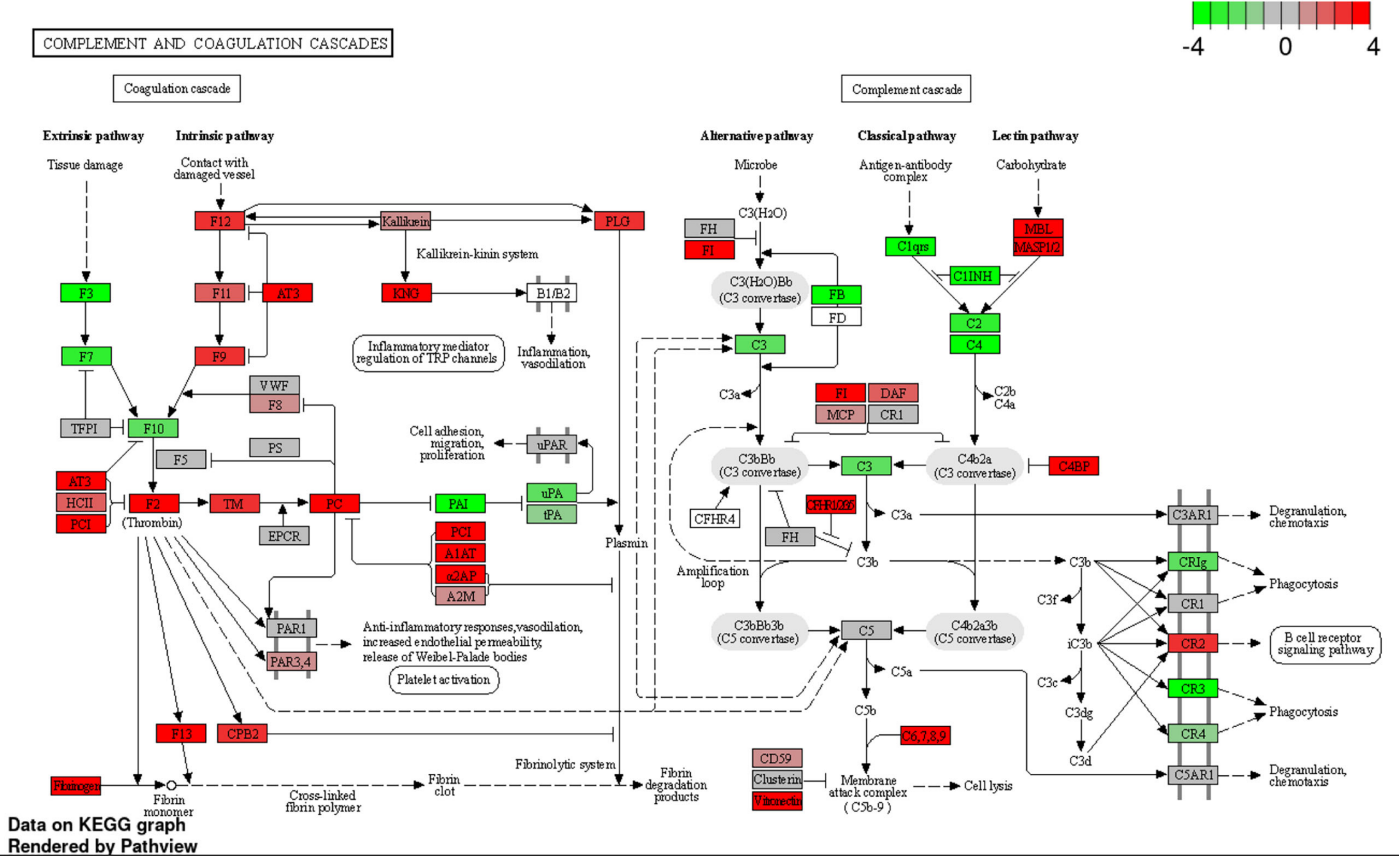
Expression profiles of complement and coagulation cascades-related genes at 36 h after infection were visualized on a KEGG pathway diagram using RNA-seq data and the iDEP Pathview package. Red and green indicate genes induced or suppressed by Δ*bgaA*-infection compared with those by WT-infection, respectively. Fold-change (log2) cutoff in color code is 4.

To investigate the effect of BgaA on the transcriptional response of host cells, leukocyte RNA was obtained from the blood of mice 12, 24, and 36 h after intravenous administration of WT or Δ*bgaA* pneumococcal strains, or PBS. The transcriptional responses of the murine leukocytes were visualized using a heat map, scatter plots, and principal component analysis (**Figures 4A, B**, and **Supplementary Figure 1**). The WT- and Δ*bgaA-*infected mice showed larger differences at later time points, while all mock mice showed similar tendencies. Venn diagrams showed that WT and Δ*bgaA-*infected mice shared 22 upregulated and 258 downregulated genes compared with PBS-treated mice at 12 h after infection (**Supplementary Figure 1**). At 24 h after infection, WT and Δ*bgaA-*infected mice shared 74 upregulated and 84 downregulated genes compared with PBS-treated mice. Moreover, WT-infected mice presented 15 additional upregulated genes and 53 additional downregulated genes compared with Δ*bgaA-*infected mice (**Figure 4B** and **Supplementary Figure 1**). At 36 h after infection, WT and Δ*bgaA-*infected mice shared 165 upregulated and 6 downregulated genes compared with PBS-treated mice. WT-infected mice presented upregulated 234 genes and downregulated 382 genes compared with Δ*bgaA-*infected mice (**Figure 4B** and **Supplementary Figure 1**). Pathway analysis of the differentially expressed genes revealed that several signaling pathways associated with host innate immunity were significantly downregulated in WT-infected mice compared with Δ*bgaA-*infected mice at 24 h and 36 h, whereas there were no significant up- or down-regulated pathways at 12 h post-infection (**Figure 4C**). Expression profiles of TNF-α signaling-related genes at 24 and 36 h after infection were visualized on a KEGG pathway diagram (**Supplementary Figure 2**). Genes encoding cytokines and chemokines, including TNF-α, CCL-2/MCP-1, and so on, were down-regulated in WT-infected mice compared with Δ*bgaA-*infected mice, while the expression of TNF-α signaling-related genes was not so largely changed. In addition, Reactome pathway analysis indicated that WT-infected mice presented significantly upregulated platelet activation pathways compared with Δ*bgaA-*infected mice at 36 h (**Supplementary Table 1**). Expression profiles of complement and coagulation cascade at 36 h after infection were also visualized on a KEGG pathway diagram (**Figure 5**). Although the complement and coagulation cascades were not significantly upregulated in WT-infected mice compared with Δ*bgaA-*infected mice, many genes were upregulated in the coagulation cascade. These results indicate that BgaA inhibits host innate immunity activation and induces coagulation cascades at the RNA level.

### BgaA Contributes to Blood Coagulation in a Mouse Sepsis Model

**Figure 6:**
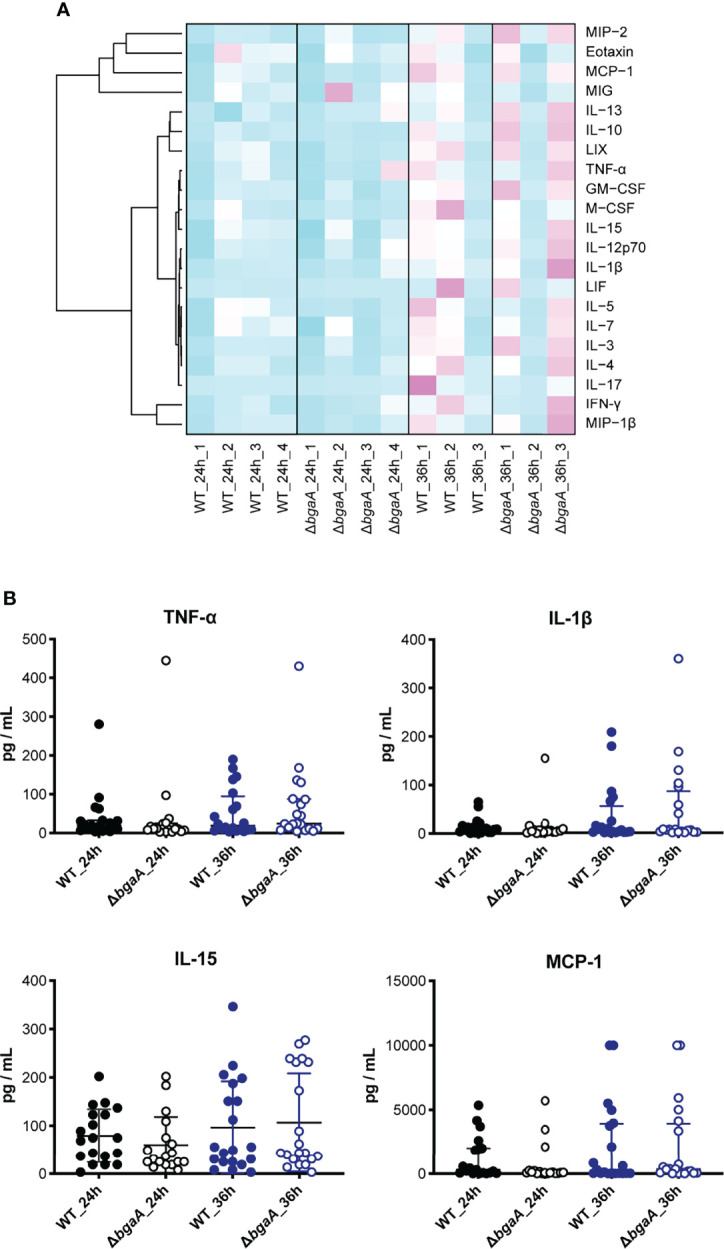
Dendrogram and heatmap of cytokine and chemokine amounts in plasma from TIGR4-infected mice 24 and 36 h after intravenous infections. **(A)** C57BL/6 mice were infected with *S. pneumoniae* TIGR4 intravenously, and the plasma was isolated 24 and 36 h after the infection. Cytokine and chemokine amounts in plasma were measured using MAGPIX. Light blue: low cytokine values; white: average values; magenta: high values. The data shown are the mean values for mice under each experimental condition (n = 3: 24h_1, 24h_2 and 36h_1, n = 6-7: 24h_3 and 24h_4, and n = 8-9: 36h_2 and 36h_3). **(B)** Actual amounts of TNF-α, IL-1β, IL-15, and MCP-1 in **(A)** All individual values were plotted. The median and IQR values are represented using vertical lines. Statistical differences between groups were analyzed using a Kruskal–Wallis test followed by Dunn’s multiple comparisons test. The data were pooled from three or four independent experiments. Other actual amounts are shown in **Supplementary Figure 3**.

**Figure 6A** shows a heatmap of the data of measured mouse cytokines and chemokines in plasmas obtained using the MAGPIX multiplexing system. The graphs of each cytokine or chemokine are shown in **Figure 6B** and **Supplementary Figure 3**. Although genes encoding TNF-α, IL-1β, IL-15, LIF, CCL-2/MCP-1, CXCL2/MIP-2, M-CSF/CSF-1, and GM-CSF/CSF-2 were down-regulated in RNA-seq analysis (**Supplementary Figure 2**), no significantly different cytokines and chemokines were observed between WT- and Δ*bgaA-*infected mice at 24 h and 36 h. These results indicate that BgaA deficiency activates host innate immunity pathways at the RNA level but not the protein level.

**Figure 7:**
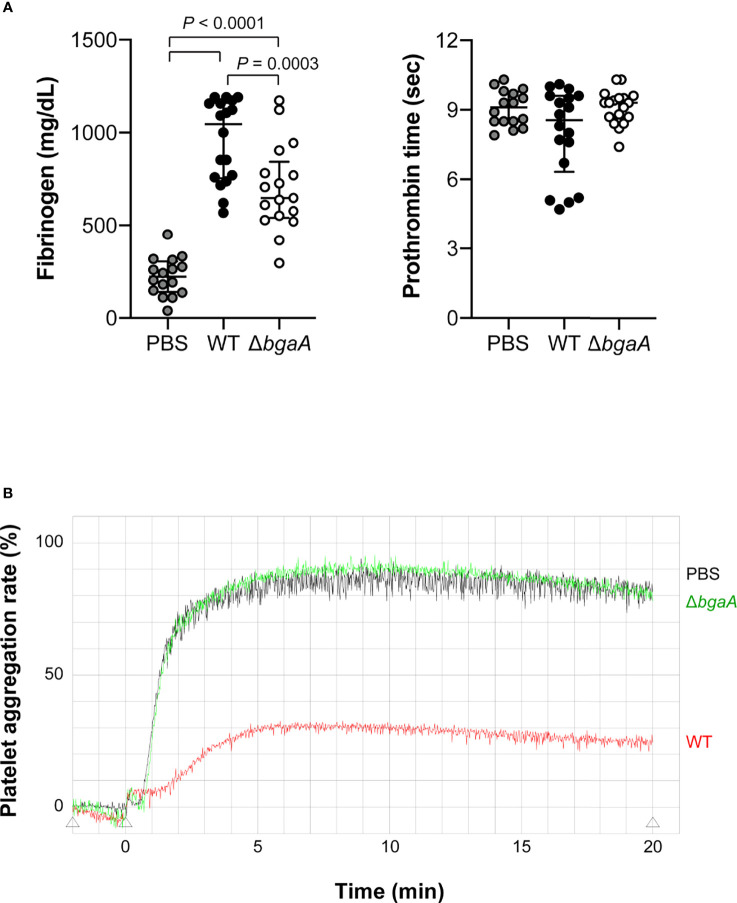
Deficiency of *bgaA* decreases fibrinogen amount in plasma and suppresses platelet aggregation rate in a mouse model of sepsis. **(A)** Fibrinogen amount in mouse plasma and prothrombin time measured by semi-automated coagulation analyzer KC1 Delta. The median and IQR values are represented using vertical lines. Statistical differences between groups were analyzed using a Kruskal–Wallis test followed by Dunn’s multiple comparisons test. The data were pooled from three independent experiments. Individual values are shown in **Supplementary Data 3**. **(B)** Platelet aggregation assessed by light transmission. The data was obtained from pooled plasma from 6 mice in each group. Data from individual plasma are shown in **Supplementary Figure 4**. Mouse blood was collected at 36 h after intravenous infection with *S. pneumoniae* TIGR4 WT and Δ*bgaA* strains **(A, B)**.

Generally, bacterial infections increase the blood concentration of fibrinogen and activate coagulation pathways (Davalos and Akassoglou, 2012). To compare blood coagulation, we measured fibrinogen levels and prothrombin time in mouse blood samples obtained 36 h after infection. WT-infected mice showed higher fibrinogen levels than the Δ*bgaA-*infected and PBS-treated mice; however, no significant differences were observed in prothrombin time among the groups of mice (**Figure 7A**). Next, we performed a platelet aggregation assay using mouse PRP and PPP 36 h after infection. As shown in **Figure 7B**, we assessed platelet aggregation rates using pooled PRP and PPP from six mice from each group. WT*-*infected mice showed reduced platelet aggregation rates compared with those in the other groups, while the level of platelet aggregation of PBS-treated and Δ*bgaA-*infected mice was comparable. In addition, we assessed platelet aggregation rates using individual PRPs and PPPs from six mice (**Supplementary Figure 4**). The individual data showed a tendency similar to that of pooled plasmas. To determine whether BgaA affects platelets directly, we performed an *in vitro* platelet aggregation assay using uninfected mouse PRPs and *S. pneumoniae* WT, Δ*bgaA*, and Δ*bgaA* with rBgaA (**Supplementary Figure 5**). The assay showed *S. pneumoniae* strains and rBgaA did not affect platelet aggregation in the PRP. These results indicate a possibility that BgaA does not act directly on platelets but inhibits the aggregation ability *via* host endothelial cells or other reactions in pneumococcal sepsis.

## Discussion

In a previous study, we demonstrated that BgaA is an evolutionarily conserved pneumococcal cell surface protein that functions as a virulence factor after mouse intravenous infection (Yamaguchi et al., 2020). In this study, we investigated the role of BgaA in pneumococcal sepsis. The deletion of *bgaA* significantly reduced pneumococcal association with human epithelial cells, in agreement with previous studies (King et al., 2006; Limoli et al., 2011; Singh et al., 2014). We also showed that the deletion of *bgaA* significantly reduced pneumococcal association with human endothelial cells and survival in human blood and human neutrophils. On the contrary, the deletion of *bgaA* did not affect pneumococcal survival in mouse blood, as demonstrated using an *ex vivo* model and evaluating the pneumococcal burden in the blood and organs of intravenously infected mice. RNA-seq enrichment pathway analysis indicated that the presence of BgaA significantly downregulated several host innate immunity pathways. However, there were no differences in cytokines and chemokines in plasma between WT- and Δ*bgaA*-infected mice. Furthermore, histopathological analysis and blood coagulation assays showed increased bleeding and microthrombus formation in the lungs and kidneys, as well as blood coagulation in WT-infected mice compared with those in PBS-treated or Δ*bgaA*-infected mice. Taken together, these results indicate that BgaA functions as a virulence factor by increasing bleeding and blood coagulation in a mouse sepsis model.

BgaA inhibits complement component C3 deposition and, consequently, opsonophagocytosis by cleaving *N*-glycans on host glycoproteins that are involved in the complement cascade (Dalia et al., 2010). In this study, we confirmed that the deletion of *bgaA* reduced pneumococcal survival in human blood and non-opsonized human neutrophils. However, no significant difference was observed in the survival of the WT and Δ*bgaA* strains in mouse blood. Interestingly, it has been reported that in the *S. pneumoniae* strain C06_18, BgaA contributes to pneumococcal association with several human epithelial cells, but not with mouse LA-4 epithelial cells (Limoli et al., 2011). BgaA mediates adherence to human epithelial cells through the interaction between its carbohydrate-binding modules and lactose or *N*-acetyllactosamine present in the cell surface of the host (Singh et al., 2014). The discrepancy between *in vitro* assays using human cells and *in vivo* mouse models could be attributed to differences in receptors between species. Thus, in a mouse model, BgaA has only a limited effect on pneumococcal association with host epithelial cells and evasion from host neutrophil killing. Moreover, BgaA induced bleeding and microthrombus formation in organs and blood coagulation in a mouse sepsis model. Furthermore, BgaA may contribute to the pneumococcal burden *via* its ability to associate with host epithelial and endothelial cells and evade opsonophagocytic killing in human sepsis.

Recently, increasing attention has been drawn to immunothrombosis, as severe acute respiratory syndrome coronavirus 2 (SARS-CoV-2) is believed to induce this process (Bonaventura et al., 2021; Loo et al., 2021). Generally, immunothrombosis is triggered by fibrin, monocytes, neutrophils, and platelets to reduce the dissemination and survival of pathogens (Engelmann and Massberg, 2013). However, uncontrolled immunothrombosis can cause thrombo-inflammation and disseminated intravascular coagulation (DIC) through a dysregulated coagulation system, leading to microthrombosis formation. Coronavirus disease 2019 (COVID-19)-associated coagulopathy is characterized by elevated levels of fibrinogen, mildly prolonged or no change in prothrombin time, and decreased or no change in platelet counts (Bonaventura et al., 2021; Loo et al., 2021). Hence, there are some similarities in the coagulopathy between our results in the mouse infection model and COVID-19, suggesting that the control of immunothrombosis may be important in various infectious diseases.

In our platelet aggregation assay, WT-infected mice showed increased fibrinogen levels in plasma, bleeding in organs, and reduced platelet aggregation rates compared with PBS-treated and Δ*bgaA*-infected mice. These results indicate that the presence of BgaA induces uncontrolled coagulation through microthrombosis and platelet consumption. The host coagulation system activates the complement pathway *via* the classical or alternative pathway, which plays a role in fibrin formation (de Bont et al., 2019). In addition, activated complement proteins can induce NETosis, a process during which neutrophils release neutrophil extracellular traps (NETs), which can serve as a direct scaffold for both thrombus formation and complement activation (de Bont et al., 2019). Since BgaA inhibits complement deposition to pneumococcal cells (Dalia et al., 2010), infection-induced excess complement might contribute to thrombus formation.

Pneumococcal NanA induces thrombocytopenia through platelet desialylation and haptic Ashwell receptor recognition (Grewal et al., 2008). In addition, NanA increases complement- and pneumolysin-mediated hemolysis and platelet aggregation (Syed et al., 2019). In vertebrates, sialic acid molecules are commonly found to be linked to galactose, *N*-acetylgalactosamine, or another sialic acid through α-glycosidic bonds (Varki et al., 2015). BgaA inhibits complement deposition by cleaving *N*-glycans on host glycoproteins involved in the complement cascade, as well as NanA and StrH (Dalia et al., 2010). Further studies are needed to determine whether BgaA can enhance thrombocytopenia by cleaving galactose residues exposed by NanA-desialylation.

This study has several limitations. First, we could not construct and use the BgaA-complemented strain. Thus, we could not exclude the possibility that some phenotypes were independent of BgaA. However, the phenotype, association with human epithelial cells, is consistent with previous studies using several strains (King et al., 2006; Dalia et al., 2010; Limoli et al., 2011). In addition, BgaA deficiency did not affect bacterial survival in *ex vivo* mouse blood and bacterial burdens in infected mouse organs. These results indirectly support the reliability of the results. Another limitation is that the precise molecular mechanism of BgaA in evasion from non-opsonic killing and the promotion of host coagulation was not elucidated. At least in PRP alone, *S. pneumoniae* and rBgaA do not affect platelet aggregation. Thus, it is possible that they affect other complex cell-mediated mechanisms to alter the coagulation response. Moreover, RNA-seq analysis of leukocytes showed that a group of genes related to the coagulation cascade was upregulated in the presence of BgaA. Although clarification of these points requires comprehensive analysis, including host ligand or receptor identification, the investigation would be able to contribute to the control of pneumococcal sepsis-induced DIC.

Our previous evolutionary analysis indicated that *bgaA*, among other cell wall-anchoring proteins, was conserved in pneumococcal species and had a high percentage of codons that were under negative selection pressure. In addition, evolutionarily conserved residues contribute to the conformation of the BgaA active site (Yamaguchi et al., 2020), and additional bioinformatic analyses also support the importance of BgaA. Prasasty *et al.* predicted host-pathogen protein-protein interactions (HP-PPIs) using a logistic regression model with HP-PPIs of three different pathogens as training data. In the predicted HP-PPI networks, network topology analysis revealed BgaA to be the most central among the *S. pneumoniae* proteins (Prasasty et al., 2021). In this study, we demonstrated that BgaA contributes to thrombus formation in a mouse sepsis model, in addition to adherence to host epithelial and endothelial cells and evasion from neutrophil-killing in humans. Our previous BLAST search indicated that BgaA does not have a high similarity with human proteins (Yamaguchi et al., 2020). Thus, BgaA could be an attractive target for new drug and vaccine development. As BgaA is a large protein containing multiple domains, it is necessary to identify the functional domain and verify its effectiveness as a drug target in further studies.

## Data Availability Statement

The datasets presented in this study can be found in online repositories. The names of the repository/repositories and accession number(s) can be found below: https://www.ncbi.nlm.nih.gov/geo/, GSE190418.

## Ethics Statement

The studies involving human participants were reviewed and approved by the institutional review board of Osaka University Graduate School of Dentistry (H26-E43). The patients/participants provided their written informed consent to participate in this study. The animal study was reviewed and approved by the Animal Care and Use Committee of Osaka University Graduate School of Dentistry (28-002-0).

## Author Contributions

MY and SK designed the study. MT and MK performed the experiments. DO and DM performed the next-generation sequencing. MY, MK, MO, and DO performed the bioinformatics analyses. MY, TS, YH, KG, MN, and SK contributed to the experimental setup. MT drafted the original manuscript. MY substantially revised the text and figures. MK, TS, YH, DO, MO, DM, KG, MN, NU, and SK contributed to the writing of the manuscript. All authors contributed to the article and approved the submitted version.

## Funding

This study was partly supported by AMED (JP20wm0325001), the Japan Society for the Promotion of Science KAKENHI (grant numbers 17H05103, 19H03825, 19K22710, 20KK0210, 20K23053, and 20K21675), SECOM Science and Technology Foundation, MSD Life Science Foundation, Public Interest Incorporated Foundation, Takeda Science Foundation, Naito Foundation, Kobayashi International Scholarship Foundation, and the Drug Discovery Science Division, Open and Transdisciplinary Research Initiatives, Osaka University. The funders had no role in the study design, data collection or analysis, decision to publish, or preparation of the manuscript.

## Conflict of Interest

The authors declare that the research was conducted in the absence of any commercial or financial relationships that could be construed as a potential conflict of interest.

## Publisher’s Note

All claims expressed in this article are solely those of the authors and do not necessarily represent those of their affiliated organizations, or those of the publisher, the editors and the reviewers. Any product that may be evaluated in this article, or claim that may be made by its manufacturer, is not guaranteed or endorsed by the publisher.
