# Supplementary material for: Pneumococcal BgaA Promotes Host Organ Bleeding and Coagulation in a Mouse Sepsis Model
Source: Front Cell Infect Microbiol. 2022 Jul 1;12:844000. doi: 10.3389/fcimb.2022.844000 (PMC9284207; doi:10.3389/fcimb.2022.844000)
Supplement: Supplementary file 1 [file DataSheet_1.zip › Supplements/Supplementary information.PDF]

Supplementary Figures

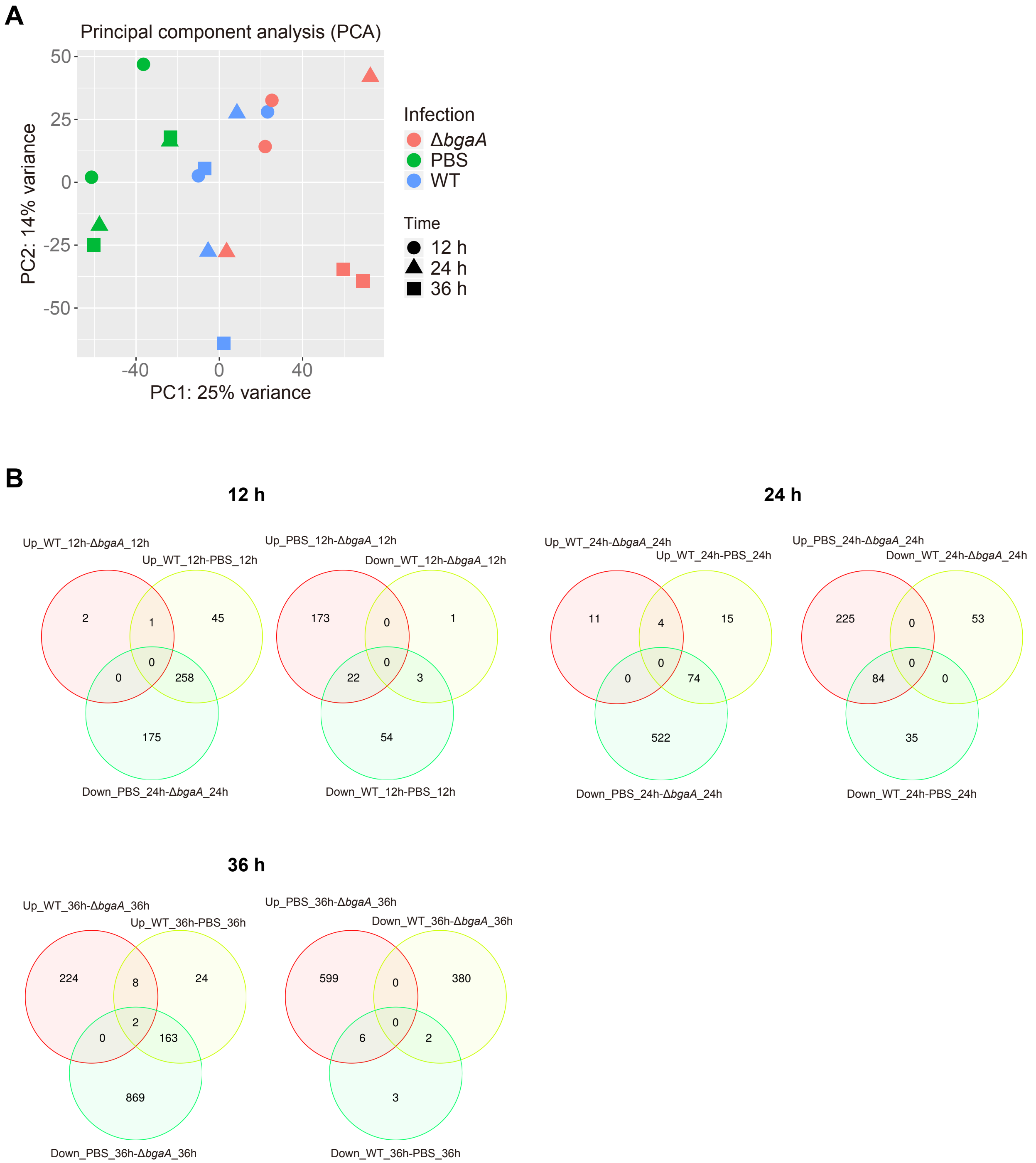

Supplementary Figure 1. (A). Principal component analysis. The color and shape represent infectious agents and time points individually. **(B).** Three-way Venn diagram illustrating host genes consistently altered among PBS-treated, wild type (WT)-infected, and Δ*bgaA*-infected mice at 12 h, 24 h and 36 h after infection. Differentially expressed genes were calculated using DESeq2. FDR cutoff value was 0.1, and the Min fold change was 2.

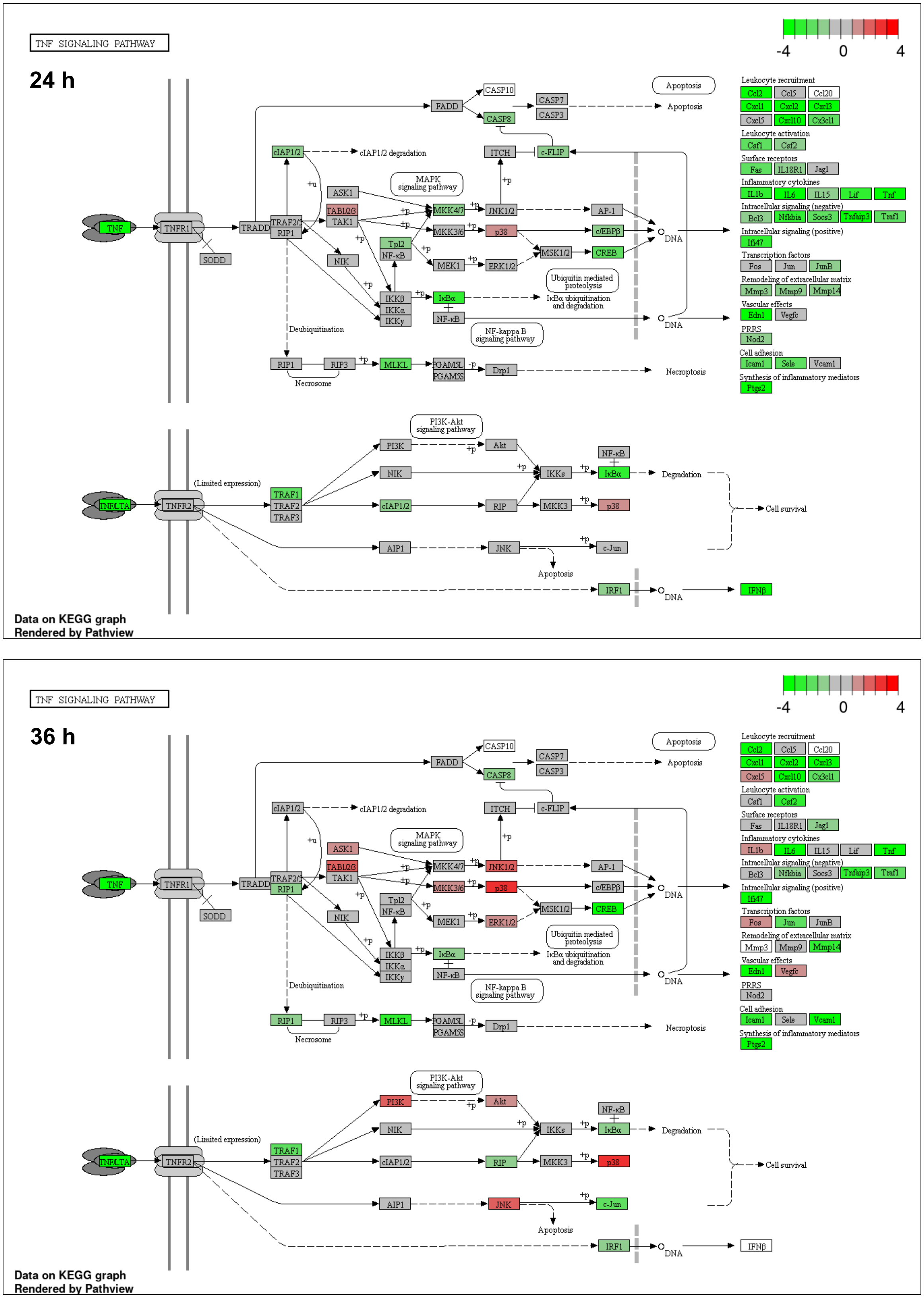

Supplementary Figure 2. Expression profiles of TNF-α signaling-related genes at 24 and 36 h after infection were visualized on a KEGG pathway diagram using RNA-seq data and the iDEP Pathview package. Red and green indicate genes induced or suppressed by Δ*bgaA*-infection as compared to the WT-infection, respectively. Fold-change (log2) cutoff in color code is 4.

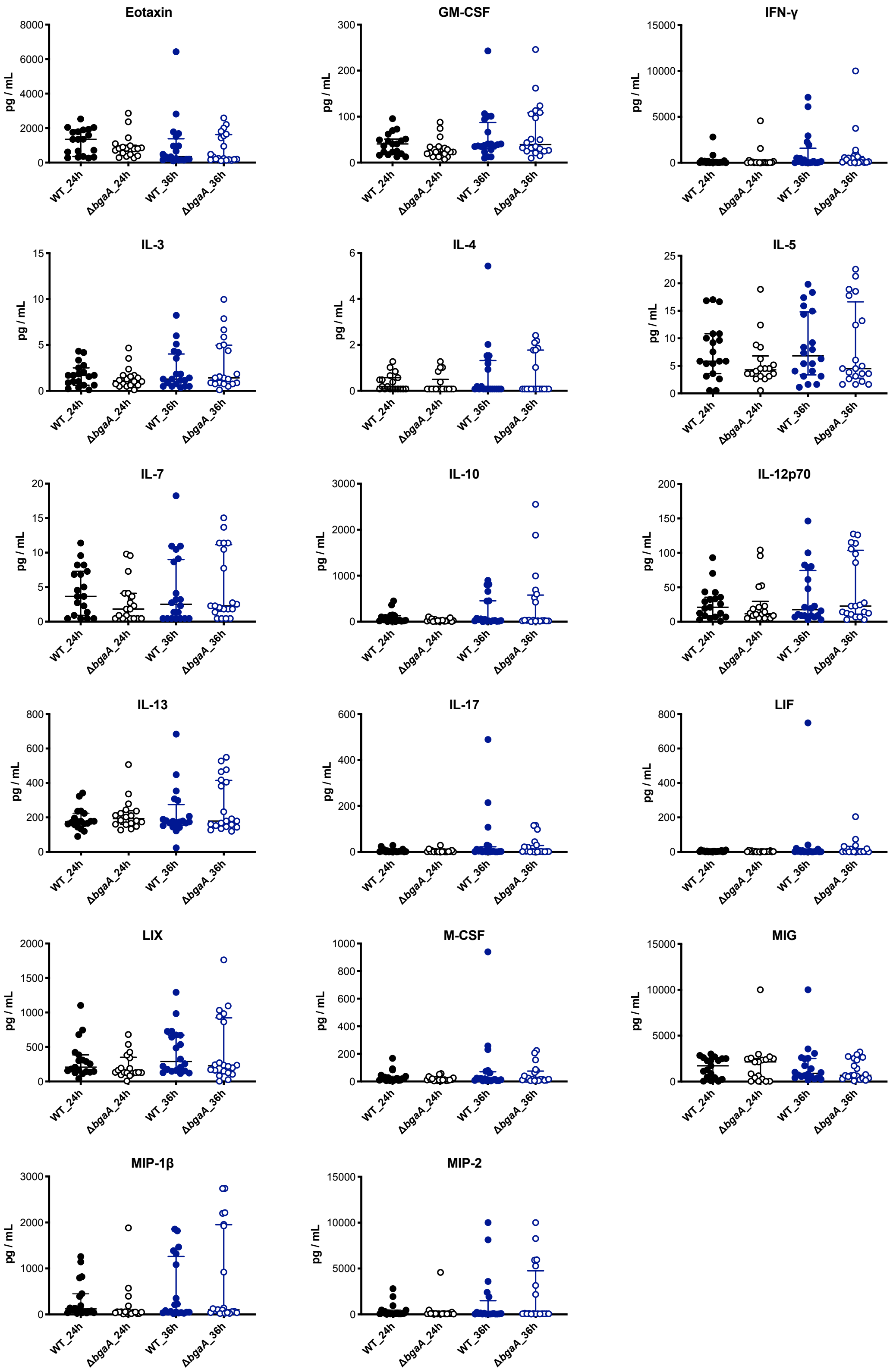

Supplementary Figure 3. Cytokine and chemokine amounts in plasma from TIGR4-infected mice at 24 and 36 h after intravenous infections. All individual values were plotted. The median and IQR values are represented using vertical lines. Statistical differences between groups were analyzed using a Kruskal–Wallis test followed by Dunn's multiple comparisons test. The data were pooled from three or four independent experiments.

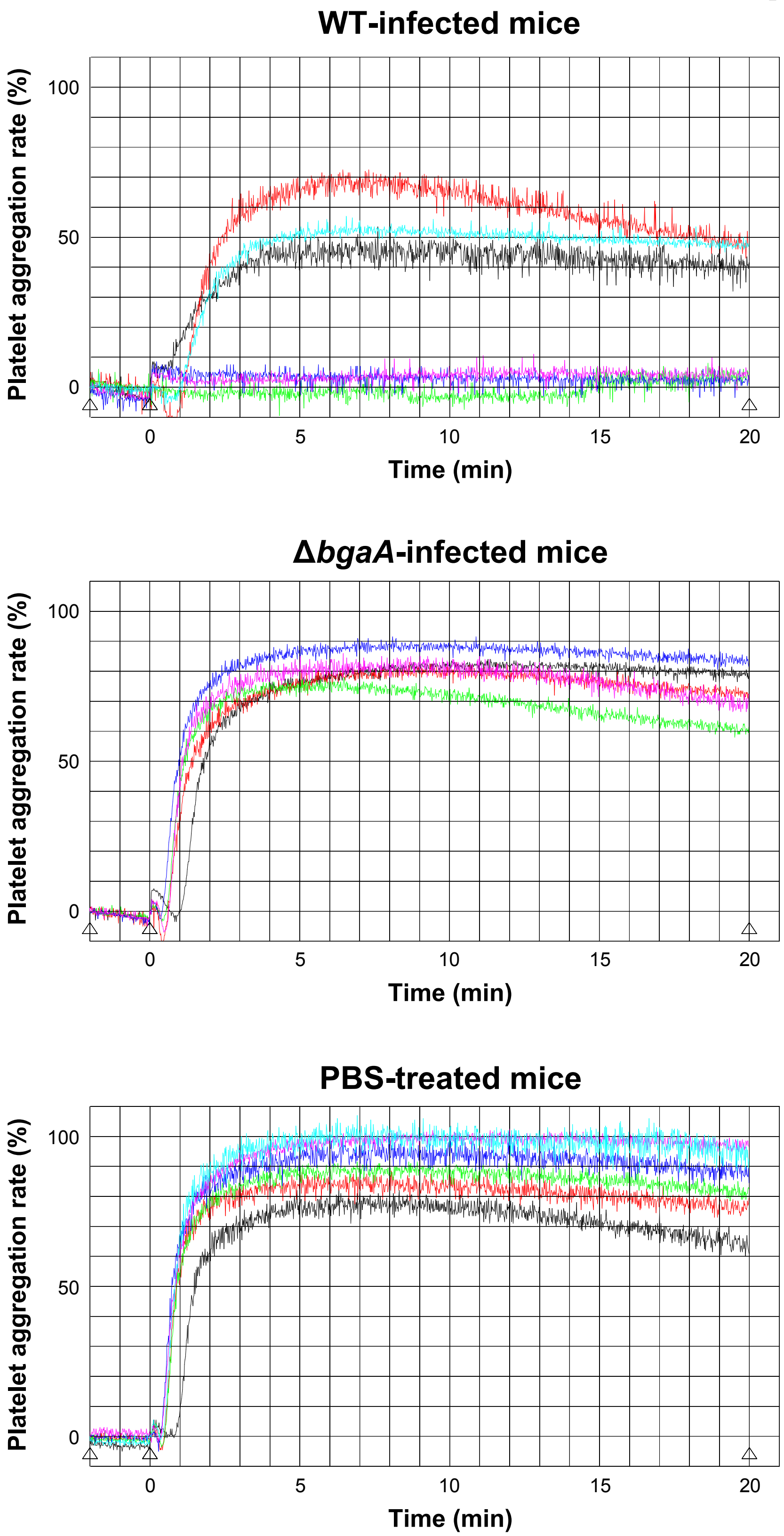

Supplementary Figure 4. Platelet aggregation assessed based on light transmission. The data were obtained from individual plasma from six mice in each group. Each line represents a different individual mouse. Mouse blood was collected at 36 h after intravenous infection with *S. pneumoniae* TIGR4 WT and Δ*bgaA* strains.

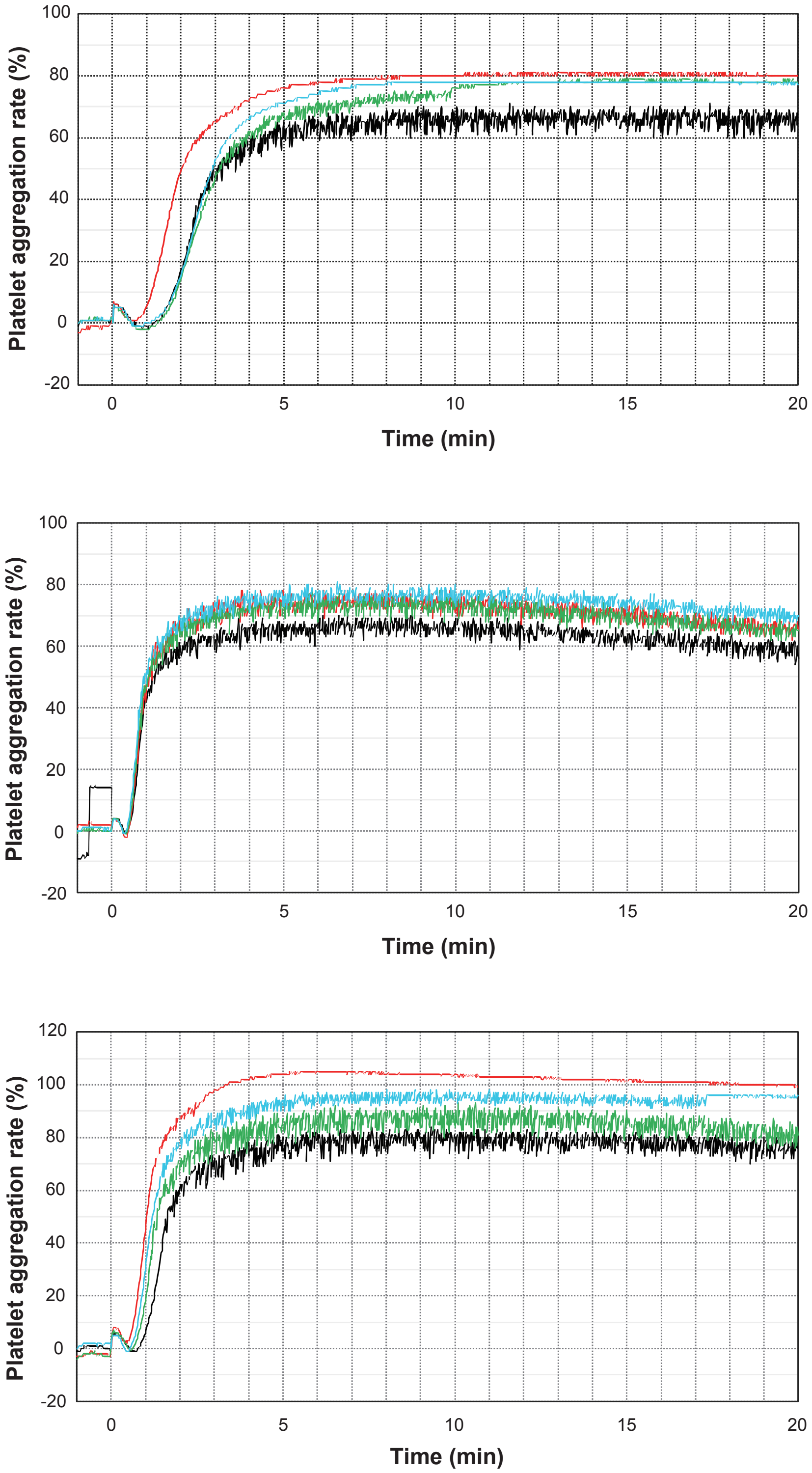

Supplementary Figure 5. Platelet aggregation assessed based on light transmission. Data are shown from 3 independent experiments. Mouse blood was immediately collected after euthanasia, and platelet rich plasma (PRP) was obtained after centrifuging the blood samples. Each bacterium was incubated in PRP for 30 min. Each sample was tested for normal aggregation responses to collagen (5 μg/mL). The change in light transmission was recorded and compared with that of autologous PPP, which was considered 100% of light transmission. Red, green, light blue, and black represents *S. pneumoniae* TIGR4 WT, Δ*bgaA*, Δ*bgaA* with rBgaA, and PBS, respectively.

Supplementary Table 1. Pathway enrichment analysis on mouse blood RNA-seq data using GAGE and Reactome pathways.

Direction	ReactomePA analysis: WT_36h vs Δ*bgaA*_36h	NES	Genes	adj.P_{val}
Down	Cell Cycle Checkpoints	-2.4344	250	3.50E-03
	Amplification of signal from the kinetochores	-2.3759	85	3.50E-03
	Amplification of signal from unattached kinetochores via a MAD2 inhibitory signal	-2.3759	85	3.50E-03
	Mitotic Spindle Checkpoint	-2.3677	101	3.50E-03
	DNA Replication	-2.3591	121	3.50E-03
	G1/S Transition	-2.3289	101	3.50E-03
	Separation of Sister Chromatids	-2.3256	170	3.50E-03
	Resolution of Sister Chromatid Cohesion	-2.3199	107	3.50E-03
	Synthesis of DNA	-2.3114	113	3.50E-03
	DNA Replication Pre-Initiation	-2.3084	81	3.50E-03
	Resolution of D-Loop Structures	-2.294	32	3.50E-03
	Cell Cycle, Mitotic	-2.2754	439	3.50E-03
	Resolution of D-loop Structures through Holliday Junction Intermediates	-2.2601	31	3.50E-03
	Mitotic Metaphase and Anaphase	-2.2561	181	3.50E-03
	Cell Cycle	-2.247	499	3.50E-03
	APC/C-mediated degradation of cell cycle proteins	-2.2461	81	3.50E-03
	Regulation of mitotic cell cycle	-2.2461	81	3.50E-03
	Mitotic Anaphase	-2.242	180	3.50E-03
	S Phase	-2.201	138	3.50E-03
	Activation of the pre-replicative complex	-2.1953	30	3.50E-03
	Mitotic G1-G1/S phases	-2.1845	123	3.50E-03
	Resolution of D-loop Structures through Synthesis-Dependent Strand Annealing (SDSA)	-2.1825	25	3.50E-03
	Activation of APC/C and APC/C:Cdc20 mediated degradation of mitotic proteins	-2.1761	74	3.50E-03
	The role of GTSE1 in G2/M progression after G2 checkpoint	-2.1729	70	3.50E-03
	Mitotic Prometaphase	-2.1591	178	3.50E-03
	APC/C:Cdc20 mediated degradation of mitotic proteins	-2.1432	73	3.50E-03
	RHO GTPases Activate Formins	-2.1432	117	3.50E-03
	Nuclear Pore Complex (NPC) Disassembly	-2.1391	30	3.50E-03
Up	Response to elevated platelet cytosolic Ca^{2+}	2.1681	103	4.00E-03
	Platelet degranulation	2.1657	98	4.00E-03

NES: Normalized enrichment scores, adj.Pval: P-values adjusted by Benjamini−Hochberg method
